# Efficacy and limitations of diffusion-weighted imaging with background suppression-based whole-body magnetic resonance imaging in high-risk prostate cancer staging: A retrospective study

**DOI:** 10.1371/journal.pone.0354601

**Published:** 2026-07-23

**Authors:** Nobuyuki Nakajima, Toshiki Kazama, Taro Takahara, Kazuya Oda, Takahiro Ogawa, Meiko Aoki, Sena Ono, Jun Naruse, Kumpei Takahashi, Soichiro Yuzuriha, Tatsuya Otaki, Tatsuya Umemoto, Masayoshi Kawakami, Yoshiaki Kawamura, Sunao Shoji

**Affiliations:** 1 Department of Urology, Tokai University School of Medicine, Isehara, Kanagawa, Japan; 2 Department of Diagnostic Radiology, Tokai University School of Medicine, Isehara, Kanagawa, Japan; 3 Department of Biomedical Engineering, Tokai University School of Engineering, Isehara, Kanagawa, Japan; 4 Department of Urology, Kouga Hospital, Yaizu, Shizuoka, Japan; Fondazione Policlinico Universitario Agostino Gemelli IRCCS, ITALY

## Abstract

**Purpose:**

To evaluate the diagnostic performance and limitations of whole-body magnetic resonance imaging (WB-MRI) using diffusion-weighted imaging with background body signal suppression (DWIBS) for staging high-risk prostate cancer (PCa) in comparison with conventional computed tomography (CT) and bone scintigraphy (BS).

**Materials and methods:**

This retrospective study included 38 patients with newly diagnosed high-risk PCa who underwent CT, bone scintigraphy (BS), and DWIBS, all performed within two months prior to treatment initiation. DWIBS was performed with coverage from the skull base to the proximal femur. Pathological findings from extended pelvic lymph node dissection were available in 14 patients and were used as the reference standard for lymph node metastasis. For all remaining cases and metastatic sites, the reference standard was established by the best valuable comparator consensus of two radiologists and two urologists using imaging and clinical follow-up data.

**Results:**

For lymph node metastases, both DWIBS and CT demonstrated a sensitivity of 64.3% and a specificity of 100.0%. For bone metastases, DWIBS showed a sensitivity of 88.9% and specificity of 100.0%, whereas BS showed a sensitivity of 100.0% and a specificity of 96.6%. Lung metastases were observed in three patients. Chest CT detected all lesions, whereas DWIBS detected two cases; these were confirmed on coronal T2-weighted images but not visualized on diffusion-weighted imaging alone.

**Conclusion:**

DWIBS demonstrated acceptable diagnostic performance for staging high-risk PCa, comparable to CT and BS, while offering the advantage of radiation-free WB-MRI and avoidance of radioisotopes. When combined with chest CT, this approach may represent a practical and feasible alternative imaging strategy for initial PCa staging.

## Introduction

In 2022, prostate cancer (PCa) was ranked as the second most common cancer globally and the fifth leading cause of cancer-related mortality among men [1]. Although PCa is increasingly detected at an early stage, 10–15% of cases continue to present with locally advanced or metastatic disease [1,2]. Therefore, accurate staging is essential for optimizing treatment selection and predicting patient outcomes, particularly in high-risk PCa.

Current clinical practice guidelines, including those of the American Urological Association [3] and National Comprehensive Cancer Network [4], recommend imaging assessment for staging; however, they do not clearly specify the optimal imaging modality or anatomical coverage, resulting in heterogeneity in real-world practice.

Diffusion-weighted imaging with background body signal suppression (DWIBS), introduced by Takahara et al. [5], enables whole-body magnetic resonance imaging (WB-MRI) without the use of contrast agents or radiation exposure. Several studies have shown that WB-MRI can detect lymph node (LN), bone, and visceral metastases with high diagnostic accuracy [6–8]. Nevertheless, magnetic resonance imaging (MRI) and CT remain limited in detecting small LN metastases, largely because of their reliance on size criteria [9,10].

Next-generation imaging modalities, such as WB-MRI and prostate-specific membrane antigen positron emission tomography (PSMA-PET), have been increasingly discussed in recent imaging guidelines [11]; however, these approaches have not yet achieved widespread implementation owing to variability in availability and a lack of prospective validation studies.

Given these uncertainties, the present study aimed to evaluate the efficacy and limitations of WB-MRI with DWIBS for staging high-risk PCa by comparing its diagnostic performance with that of conventional Computed Tomography (CT) and bone scintigraphy (BS).

## Materials and methods

### Study design and population

**Fig 1:**
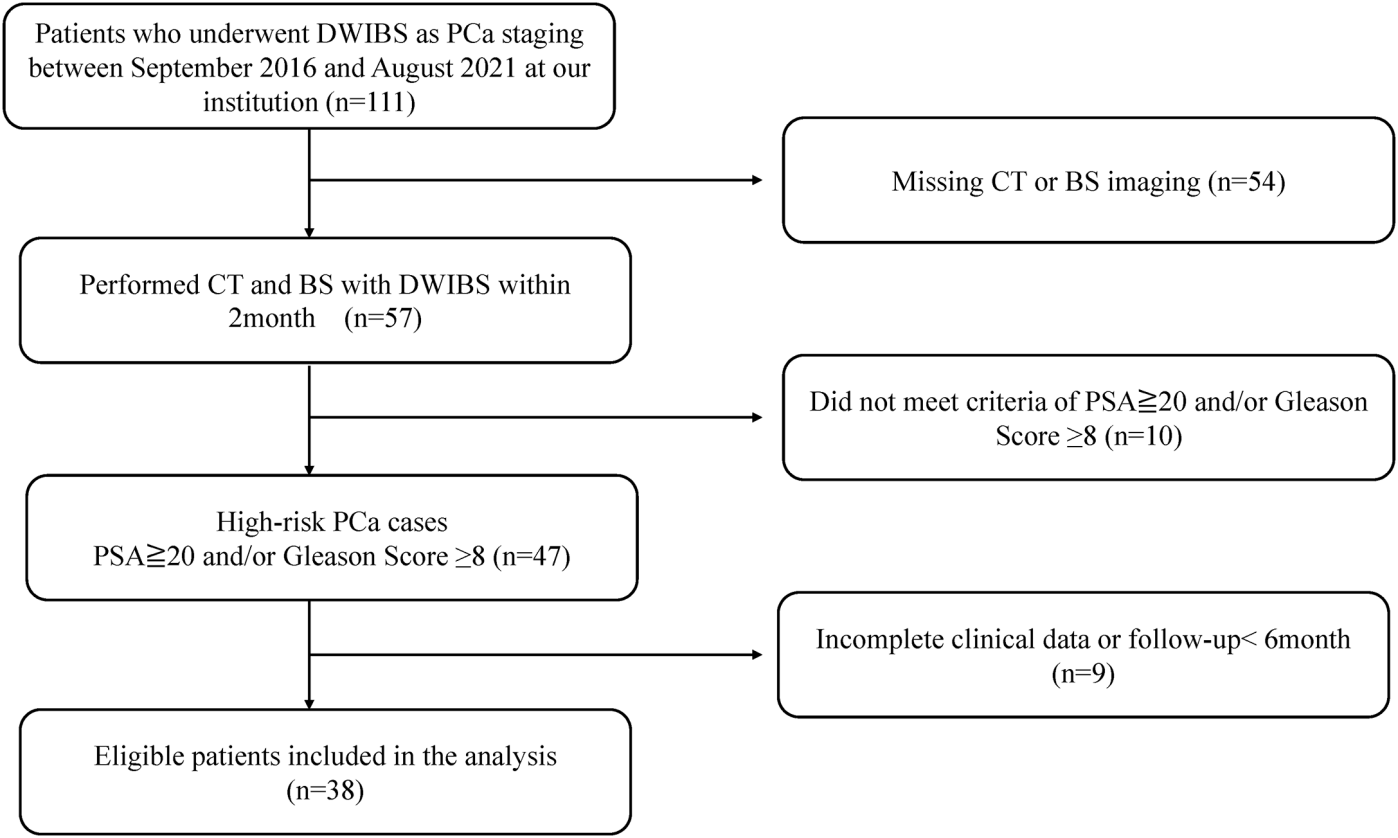
Study flow chart. From September 2016 to August 2021, 111 patients who underwent diffusion-weighted imaging with background body signal suppression (DWIBS) for staging prostate cancer (PCa) were identified. Of these, 54 patients were excluded owing to missing computed tomography (CT) or bone scintigraphy (BS) imaging, 10 patients did not meet the high-risk criteria (prostate-specific antigen [PSA] ≥20 ng/mL or Gleason score ≥8), and 9 patients were excluded owing to incomplete clinical data or follow-up <6 months. The final analysis included 38 eligible patients.

This retrospective study included 38 patients who were newly diagnosed with high-risk PCa, defined as a Gleason score of ≥8 and/or a prostate-specific antigen (PSA) level of ≥20 ng/mL. DWIBS examinations for all eligible patients were performed between November 2016 and August 2021 at our institution. Each patient underwent CT, bone scintigraphy, and DWIBS as part of primary staging, all performed within 2 months before initiation of primary treatment. Patients were followed for at least 6 months. The inclusion criteria ensured that only newly diagnosed high-risk PCa patients with complete imaging data for all three modalities were included. To ensure uniformity in the assessment of diagnostic accuracy, individuals who had previously received treatment for PCa or who lacked complete imaging data were excluded (Fig 1).

This study was approved by the local Institutional Review Board (approval number: 22R205) and was conducted in accordance with the principles of the 1964 Helsinki Declaration. The requirement for informed consent was waived because of the retrospective nature of this study. Clinical and imaging data were accessed on multiple occasions for research purposes, beginning on January 7, 2023. The authors had access to potentially identifiable patient information only during data retrieval; however, all data were anonymized prior to analysis, and no identifiable personal information was included in the final dataset.

### Imaging protocols

#### Diffusion-weighted imaging with background body signal suppression (DWIBS).

All DWIBS examinations were performed using a 3T MRI scanner (Ingenia; Philips Medical Systems) with a sliding surface coil approach without contrast agent administration. Maximum intensity projection and volumetric reconstructions, including fusion images with axial T2-weighted imaging, were used to improve lesion detection.

The detailed acquisition parameters, including TR/TE, b-values, slice thickness, acquisition matrix, number of stations, and scan time are summarized in Table 1. The total scan time was approximately 35 min, with anatomic coverage from the skull base to the proximal femur. In this study, “whole-body” DWIBS refers to this institutional imaging range rather than vertex-to–mid-thigh coverage. DWIBS images were reviewed by two experienced radiologists. Initial image assessments were performed separately, and final imaging interpretations were determined by consensus. Formal inter-reader reliability analysis was not performed, which is acknowledged as a limitation of this study. The timing of DWIBS, CT, and BS relative to treatment initiation is described in the Study design and population section.

**Table 1:** DWIBS acquisition protocol.

Parameter	DWIBS	STIR	FSE Dixon	GRE Dixon	Axial T2WI	Coronal T2WI
Imaging area	skull base to proximal femur	Whole spine	Whole spine	skull base to proximal femur	skull base to proximal femur	skull base to proximal femur
Scan orientation	Coronal	Sagittal	Sagittal	Coronal	Axial	Coronal
Dimension	2D	2D	2D	3D	2D	2D
TR/TE (ms)	6035/70	5383/60	597/10	3.6/2.4	980/85	693/90
Flip angle (degrees)	90	90	90	10	90	90
FOV (mm)	280	300	300	280	350	430
Acquisition matrix	160 × 155	436 × 215	440 × 298	291 × 257	320 × 254	400 × 320
Slice thickness/space	4.5/4.5	4/4.4	4/4.4	6/6	7/8	7/7.7
Scan technique	Single shot EPI	FSE	FSE Dixon	GRE Dixon	FSE	FSE
Fat suppression	STIR	STIR				
b-values (s/mm^2^)	0, 1000					
No. of signals acquired	2	1	1	1	1	1
No. of stations	3	2	2	3	2	2
Acquisition time (one station)	3:01	1:18	1:27	0:18	0:20	0:31

Detailed acquisition parameters of DWIBS, including the sequence, TR/TE, slice thickness, b-values, field of view, STIR settings, FSE, and GRE. Abbreviations: DWIBS, diffusion-weighted whole-body imaging with background suppression; STIR, short tau inversion recovery; FSE, fast spin echo; GRE, gradient recalled echo; TR/TE, repetition time/echo time; FOV, field of view; no., number; T2WI, T2-weighted imaging; 2D, two-dimensional.

#### Bone Scintigraphy (BS).

BS was performed by administering a bolus injection of 555-MBq technetium-99m methylene diphosphonate and performing whole-body scanning 3 hours later. An experienced nuclear medicine physician categorized the image data.

#### Computed Tomography (CT).

Multidetector chest and abdominal CT scans (with or without contrast) were performed to detect LN and visceral metastases. Two radiologists with experience in PCa staging reviewed the images to ensure the reliability of LN and visceral metastases detection. Both chest and abdominal CT scans were acquired with a slice thickness of 5 mm, which reflects the standard protocols used at our institution.

#### Reference standard (Best valuable comparator).

Owing to the lack of histological confirmation of bone and LN metastases in most cases, the best valuable comparator was employed as the diagnostic reference standard. This method, which has been validated in previous comparative imaging studies, involves a comprehensive consensus review of all available imaging and clinical follow-up data [7]. The reference standard assessment was conducted by two radiology specialists and two urologists to establish the presence or absence of metastasis based on longitudinal imaging and clinical data.

Benign cystic bone lesions were not predefined as an independent diagnostic category in this study. Bone lesions were classified dichotomously as metastatic or non-metastatic based on consensus interpretation of BS, DWIBS findings, and follow-up clinical data. Lesions without imaging or clinical features suggestive of metastasis were classified as non-metastatic.

LNs with a short-axis diameter of ≥8 mm were defined as metastatic. In addition, pathological results from extended pelvic LN dissection were used as the reference standard in 14 patients.

#### Statistical analysis.

As this was a retrospective study, no formal sample size calculation was performed. All consecutive patients who met the inclusion criteria and underwent DWIBS, CT, and BS during the study period were included in the analysis. Thus, the sample size was determined on the basis of the number of eligible cases available during the specified time frame.

For each imaging technique, sensitivity, specificity, positive predictive value (PPV), negative predictive value (NPV), and accuracy were determined based on the best valuable comparator consensus. The McNemar test was used to compare the diagnostic accuracy of the imaging modalities. Statistical significance was set at p < 0.05, and all statistical analyses were conducted using Python (version 3.13) with the relevant statistical packages.

## Results

### Patient characteristics

The median age of the 38 patients was 67 years (range, 57–79 years), and the median PSA level was 43.72 ng/mL (range, 6.76–8593.9). The Gleason score (GS) distribution was as follows: GS 6, n = 1; GS 7, n = 11; GS 8, n = 17; GS 9, n = 8; and GS 10, n = 1 (Table 2). According to the best valuable comparator, lymph node (LN) metastasis was detected in 14 cases, bone metastasis in 9, and lung metastasis in three (Tables 3 and 4).

**Table 2:** Patient background characteristics.

Patient background (N = 38)	
Age	67.0 (57-79)
PSA	43.72 (6.76-8593.9)
Gleason Score	
6	1
7	11
8	17
9	8
10	1
Treatment	
ADT	14
Surgery	14
Radiation + ADT	9
Other	1
T stage	
T2a	2
T2b	1
T2c	12
T3a	6
T3b	15
T4	2

Values are presented as median (range) or numbers (%). PSA: prostate-specific antigen.

ADT, androgen deprivation therapy.

**Table 3:** Imaging results for predicting lymph node (LN) metastases on the basis of the best valuable comparator.

		Best valuable comparator for LN metastases							
		negative n = 24	positive n = 14	total n = 38	Sensitivity (95% CI)	Specificity (95% CI)	PPV (95%CI)	NPV (95%CI)	Accuracy (95%CI)
DWIBS	negative	24	5	29	64.3 (35.1-87.2)	100.0 (79.6-100.0)	100.0 (55.5-100.0)	82.8 (64.2-94.2)	86.9 (72.7-94.3)
	positive	0	9	9					
CT	negative	24	5	29	64.3 (35.1-87.2)	100.0 (79.6-100.0)	100.0 (55.5-100.0)	82.8 (64.2-94.2)	86.9 (72.7-94.3)
	positive	0	9	9					

Sensitivity, specificity, positive predictive value, negative predictive value, and accuracy of DWIBS and CT for lymph node metastases using the best valuable comparator as the reference standard. DWIBS, diffusion-weighted imaging with background body signal suppression; CT, computed tomography.

**Table 4:** Imaging results for predicting bone metastases on the basis of the best valuable comparator.

		Best valuable comparator for Bone metastases							
		negative n = 29	positive n = 9	total n = 38	Sensitivity (95% CI)	Specificity (95% CI)	PPV (95%CI)	NPV (95%CI)	Accuracy (95%CI)
DWIBS	negative	29	1	30	88.9 (51.8-99.7)	100.0 (81.1-100.0)	100.0 (63.1-100.0)	96.7 (82.8-99.9)	97.4 (86.2-99.9)
	positive	0	8	8					
BS	negative	28	0	28	100.0 (66.4-100.0)	96.6 (82.2-99.9)	90.0 (55.5-99.7)	100.0 (87.7-100.0)	97.4 (86.2-99.9)
	positive	1	9	10					

Sensitivity, specificity, positive predictive value, negative predictive value, and accuracy of DWIBS and BS for bone metastases using the best valuable comparator as the reference standard. DWIBS, diffusion-weighted imaging with background body signal suppression; BS, bone scintigraphy.

### LN metastasis detection

The diagnostic performances of DWIBS and CT for LN metastasis were evaluated using the best valuable comparator as the reference standard. For DWIBS, the sensitivity was 64.3% (95% CI: 35.1–87.2), specificity 100.0% (95% CI: 79.6–100.0), PPV 100.0% (95% CI: 55.5–100.0), NPV 82.8% (95% CI: 64.2–94.2), and accuracy 86.9% (95% CI: 72.7–94.3). CT demonstrated an identical diagnostic performance. Both modalities correctly classified 33 of the 38 cases (9 true positives and 24 true negatives) with no discordant results. Therefore, the McNemar test could not be performed because no disagreements were observed between the DWIBS and CT results (Table 3). These findings reflect the limitations of size-based criteria, which affect both modalities equally.

### Bone metastasis detection

**Fig 2:**
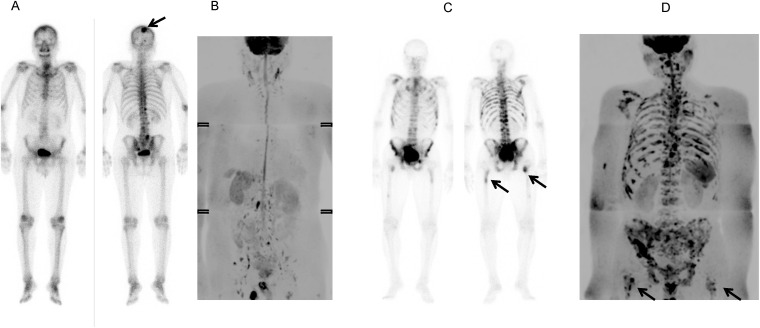
Comparison of bone scintigraphy (BS) and diffusion-weighted imaging with background body signal suppression (DWIBS) for the detection of bone metastases in two cases. (A, B) Images from the same patients. (A) BS reveals skull metastasis that could not be visualized on DWIBS (B) because it was outside the imaging range. (C, D) Images from other patients. (C) BS showing a metastatic lesion in the proximal femur, which was also clearly detected on DWIBS (D) as it was within the imaging range. These cases highlight the importance of the imaging range when evaluating skeletal metastases using DWIBS.

DWIBS demonstrated a sensitivity of 88.9% (95% CI: 51.8–99.7), specificity 100.0% (95% CI: 88.1–100.0), PPV 100.0% (95% CI: 63.1–100.0), NPV 96.7% (95% CI: 82.8–99.9), and accuracy 97.4% (95% CI: 86.2–99.9). In comparison, BS achieved a sensitivity of 100.0% (95% CI: 66.4–100.0), specificity 96.6% (95% CI: 82.2–99.9), PPV 90.0% (95% CI: 55.5–99.7), NPV 100.0% (95% CI: 87.7–100.0), and accuracy 97.4% (95% CI: 86.2–99.9). No significant difference was observed between the diagnostic accuracies of DWIBS and BS (p = 1.0) (Table 4). Among the 9 patients with bone metastasis, 7 had PSA levels ≥20 ng/mL. The PSA distribution ranged from 6.76 to 8593.87 ng/mL, showing a substantial variability. In the patient-based analysis, no differences in sensitivity or specificity were observed between DWIBS and BS. However, in the lesion-based analysis, two skull metastases detected by BS were not detected by DWIBS. This was because the skull was outside the DWIBS imaging range used at our institution (Fig 2 and Table 5). All missed lesions were located above the skull base and were outside the DWIBS acquisition field.

**Table 5:** Bone metastatic sites.

Metastatic site	Case (total, 9cases)
Spine	9 (100%)
Pelvis	9 (100%)
Ribs	4 (44.4%)
Sternum	3 (33.3%)
Femur	3 (33.3%)
Scapula	3 (33.3%)
Skull	2 (22.2%)
Other	1 (11.1%)

### Visceral metastasis detection

**Fig 3:**
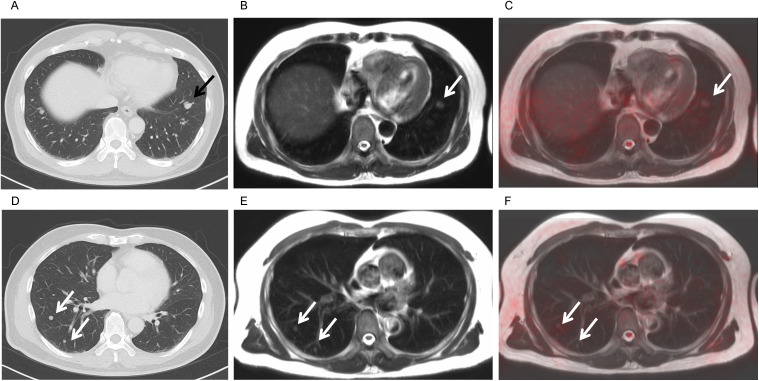
Comparison of lung metastasis detection between computed tomography (CT) and magnetic resonance imaging (MRI). Images A–C and D–F correspond to the same metastatic lung lesions. A and D: Axial chest CT clearly demonstrates lung metastases. B and E: Corresponding T2-weighted MRI shows lesions with reduced conspicuity. C and F: Fusion images of T2-weighted and diffusion-weighted imaging (DWI) failed to visualize the lesions, indicating the limited sensitivity of DWI in detecting small lung metastases.

Lung metastases were observed in three patients. CT detected all three lesions. DWIBS identified two of these three cases. In our institutional protocol, DWIBS acquisition includes diffusion-weighted imaging and coronal T2-weighted sequences, and image interpretation was based on integrated assessment of all acquired sequences. In the two detected cases, the lesions were not clearly visualized on diffusion-weighted imaging alone but were identified on the coronal T2-weighted images (Fig 3).

## Discussion

The efficacy of DWIBS for PCa staging was reported by Lecouvet et al. [7], and we aimed to confirm its efficacy while examining its limitations. Regarding the detection of LN metastasis, DWIBS and CT have perfect specificity but limited sensitivity for LN metastasis detection, resulting in a high PPV and moderate NPV. This result aligns with those of previous studies on the limitations of MRI and CT in detecting small LN metastases [9,10]. A meta-analysis published in 2008 reported pooled sensitivities of 0.42 (95% CI: 0.26–0.56) for CT and 0.39 (95% CI: 0.22–0.56) for MRI, with identical pooled specificities of 0.82 for both modalities (CT: 95% CI, 0.80–0.83; MRI: 95% CI, 0.79–0.83), indicating similarly low sensitivities of CT and MRI for LN metastasis detection. The meta-analysis further highlighted that relying solely on either CT or MRI for LN metastasis detection risks misrepresenting the true metastatic status of the patient, potentially leading to inappropriate treatment strategies [9]. One of the main reasons for the low sensitivity of CT and MRI in detecting LN metastases is their reliance on tumor size as a primary diagnostic criterion. In the present study, we used a threshold of 8 mm to identify positive LN metastases. Five of the seven cases were confirmed to have LN metastasis missed by DWIBS and CT owing to their smaller size (<8 mm). This highlights the limitations of size-based criteria in the detection of small LN metastases. Although a short diameter of 8–10 mm is often reported as the size criterion, approximately 20% of patients with PCa have LN metastases with normal diameters on preoperative imaging [12]. In their study, Thoeny et al. performed LN dissection in 120 individuals with prostate and bladder cancers. They reported that out of 88 metastatic LNs in 33 patients, 68 nodes measured ≤3 mm, while only two nodes were >8 mm [13]. Although reducing the size threshold has been explored, studies have shown that lowering this criterion does not consistently improve diagnostic accuracy [14]. Techniques such as DWI and apparent diffusion coefficient values have been suggested to improve the diagnostic capabilities of MRI, although there is no consensus on their use for routine staging [15]. Compared with MRI and CT, PSMA-PET has a higher sensitivity (77–100%) for detecting LN metastases [16–18]. However, PSMA-PET is not reliable for detecting small LN metastases [19], and the most reliable method for detecting LN metastases is extended pelvic LN dissection [9]. Additionally, PSMA-PET presents accessibility issues, as reliance on specific tracers and advanced equipment may limit their availability, particularly in regions with limited resources. These factors can pose challenges for widespread implementation in some institutions. In contrast, DWIBS offers a radiation-free whole-body imaging approach with widely available MRI systems. Understanding the respective advantages and limitations of DWIBS and PSMA-PET is essential. These imaging modalities may be considered complementary, depending on the clinical context and resource availability.

In our study, the diagnostic performances of DWIBS and BS for detecting bone metastases were comparable; however, previous studies have reported that DWIBS outperforms BS in terms of bone metastasis detection [7,8] without radiation exposure. This advantage is particularly important for patients requiring frequent imaging. WB-MRI can detect lesions earlier than BS because it can identify metastatic cancer cells in the bone marrow cavity before bone remodeling occurs [20,21]. Our study showed that the median PSA level among patients with bone metastases was 240.4 (6.76–8593.87) ng/mL, and 7 of 9 patients (77.8%) had a PSA level of ≥20 ng/mL, supporting the role of PSA as a biomarker to guide staging evaluations and imaging choices in high-risk cases [3]. Most bone metastases predominantly involve the axial skeleton, such as the spine and pelvic bones [22]. However, in two cases, parietal bone metastases were identified only on BS images (Fig 2). Because these lesions were located above the skull base and therefore outside the imaging range of our DWIBS protocol, they were not detected. Although skull metastases are rare in other cancers, they are common in prostate and breast cancer [22]. Additionally, femoral metastases were observed in three cases, all of which were in the proximal femur and successfully detected using DWIBS. In 2016, Padhani et al. proposed the METastasis Reporting and Data System for Prostate Cancer (MET-RADS-P) guidelines, which standardized imaging techniques and evaluation methods for WB-MRI in patients with advanced PCa [23]. According to the MET-RADS-P guidelines, the imaging range of DWIBS should ideally cover the region from the vertex to proximal femur. In our study, the DWIBS range (skull base to proximal femur) did not fully conform to this recommendation, which may have contributed to missed skull lesions located outside the imaging field, particularly in the skull above the skull base.

Regarding lung metastasis detection, our study showed that DWIBS identified lung metastases in two of three cases (66.7%), highlighting its limitations in detecting smaller lesions, particularly those near the heart. In comparison, chest CT is superior for the detection of lung metastases [24]. Postmortem analyses have reported that approximately 46% of patients with PCa demonstrate lung metastases; however, patients with lung-only metastases without concurrent bone or visceral metastases account for only 4.6% of the cases [25,26]. The positivity rates of lung and spinal metastases were not correlated, and lung metastases are considered to be associated with dissemination via the inferior vena cava rather than the backward venous spread [26]. This may need to be included in comprehensive thoracic evaluations for high-risk PCa staging in the future.

Based on these findings, our institutional DWIBS protocol, which covered the region from the skull base to the proximal femur, may have contributed to missed cranial lesions. Therefore, DWIBS with extended coverage from the vertex to the mid-thigh, in accordance with MET-RADS-P recommendations, combined with chest CT, may provide a more comprehensive staging approach for high-risk PCa. This strategy allows radiation-free whole-body imaging and avoids the use of radioisotopes.

The limitations of this study include its retrospective design and its limited sample size, which resulted in wide confidence intervals for some diagnostic performance estimates. Therefore, these estimates should be interpreted with caution and validated in larger prospective cohorts. In addition, histopathological confirmation of metastases was not available for most patients, particularly for bone metastases; therefore, the best valuable comparator based on consensus interpretation of imaging findings and clinical follow-up data was used as the reference standard. Because the best valuable comparator incorporated information from the index tests, incorporation bias may have occurred, potentially leading to overestimation of diagnostic performance, particularly specificity. Bone lesions were not classified into detailed benign subcategories and were dichotomously assessed as metastatic or non-metastatic, which may have influenced lesion characterization.

Inter-reader agreement was not formally assessed. Although all images were interpreted in consensus by two experienced radiologists, the lack of independent reading represents a limitation. Furthermore, although DWIBS, CT and BS were performed within a predefined time window, residual temporal differences between imaging modalities may have affected lesion detectability.

This study also focused on patient-level rather than site-specific analysis of metastases, which may have limited detailed lesion-based comparison.

## Conclusions

The combination of DWIBS and chest CT may provide acceptable diagnostic performance for staging high-risk PCa in this retrospective cohort, while minimizing radiation exposure and maintaining high specificity for LN and bone metastases. This imaging approach may represent a practical and feasible staging strategy, particularly in clinical settings where access to advanced molecular imaging is limited. These findings should be interpreted with caution given the retrospective design and limited sample size.

## Supporting information

S1 Dataset Anonymized minimal dataset underlying the findings of this study. (XLSX)
