# Supplementary material for: Efficacy and limitations of diffusion-weighted imaging with background suppression-based whole-body magnetic resonance imaging in high-risk prostate cancer staging: A retrospective study
Source: PLoS One. 2026 Jul 23;21(7):e0354601. doi: 10.1371/journal.pone.0354601 (PMC13395373; doi:10.1371/journal.pone.0354601)
Supplement: Attachment — Submitted filename: renamed_734af.docx [file pone.0354601.s004.docx]

**Response to Reviewer**

We sincerely thank the reviewer for the careful evaluation of our revised manuscript and for the constructive comments. We are grateful for the positive assessment that the manuscript has been substantially improved. We have carefully addressed all comments as detailed below.

**Comment 1. Data availability policy**

Reviewer comment:

The reviewer recommended providing an anonymized minimal dataset as a supplementary file to comply with the PLOS ONE open-data policy.

Response:

Thank you for this important suggestion. In accordance with the PLOS ONE data availability policy, we have prepared and included an anonymized minimal dataset as **S1 Dataset**. The dataset contains demographic information (age, PSA level, Gleason score, clinical T stage) together with the binary diagnostic outcomes for CT, bone scintigraphy, DWIBS, and the reference standard. All direct identifiers and dates have been removed to ensure patient confidentiality. The Data Availability Statement has been revised accordingly.

**Comment 2. Sample size and confidence intervals**

Reviewer comment:

The reviewer suggested explicitly stating that the small sample size resulted in wide confidence intervals and that the reported diagnostic performance estimates should be interpreted cautiously.

Response:

We appreciate this valuable suggestion. We have added the following statement to the Limitations section:

*"The limitations of this study include its retrospective design and its limited sample size, which resulted in wide confidence intervals for some diagnostic performance estimates. Therefore, these estimates should be interpreted with caution and validated in larger prospective cohorts."*

**Comment 3. Reference standard and incorporation bias**

Reviewer comment:

The reviewer suggested explicitly acknowledging the potential incorporation bias introduced by using the Best Valuable Comparator (BVC) as the reference standard.

Response:

We agree with this important methodological consideration. We have revised the Limitations section to explicitly acknowledge the possibility of incorporation bias by adding the following sentence:

*"Because the best valuable comparator incorporated information from the index tests, incorporation bias may have occurred, potentially leading to overestimation of diagnostic performance, particularly specificity."*

**Additional revisions**

During preparation of the anonymized minimal dataset, we performed a comprehensive verification of the analytic dataset. During this process, we identified an error in the reported number of bone metastases according to the best valuable comparator. After correction, the number of bone metastases was revised from 11 to 9, and the corresponding diagnostic performance metrics and related text were updated throughout the manuscript. These corrections do not alter the overall conclusions of the study.

In addition, the Methods section was revised to clarify that DWIBS examinations for the eligible patients included in the final analysis were performed between November 2016 and August 2021, consistent with the approved study period.

We also corrected wording in the Abstract and Methods section to clarify that pathological findings from extended pelvic lymph node dissection were available in 14 patients and were used as the reference standard for lymph node assessment, rather than implying that all 14 patients had pathologically confirmed lymph node metastases.

Thank you for your time and consideration.

Sincerely,

Nobuyuki Nakajima, M.D., Ph.D.

On behalf of all authors
