# Supplementary material for: Efficacy and limitations of diffusion-weighted imaging with background suppression-based whole-body magnetic resonance imaging in high-risk prostate cancer staging: A retrospective study
Source: PLoS One. 2026 Jul 23;21(7):e0354601. doi: 10.1371/journal.pone.0354601 (PMC13395373; doi:10.1371/journal.pone.0354601)
Supplement: Attachment — Submitted filename: PLOS Response to reviewer.docx [file pone.0354601.s003.docx]

**Response to Reviewers**

We thank the Editor and the Reviewers for their careful evaluation of our manuscript and for their constructive and insightful comments. We have revised the manuscript accordingly and believe that these revisions have substantially improved the methodological clarity, transparency, and internal consistency of the study. All changes have been incorporated into the revised manuscript, and the corresponding sections have been updated as indicated.

**Response to Reviewer #1**

**Comment #1**
This is a relatively small retrospective clinical study on the usefulness of DWIBS combined with chest CT to stage high-risk metastatic prostate cancer. The paper’s conclusion that “DWI plus chest CT offers an effective and balanced staging…” should include repeating several of the caveats given in the Limitations section.

**Response**
We sincerely thank the reviewer for this constructive comment. We fully agree that the conclusions should appropriately reflect the limitations inherent to this retrospective study.

Accordingly, we have revised both the Abstract (Lines37-62) and the Conclusions (Lines 390-395) section to temper the wording and explicitly reiterate the key limitations, including the retrospective design, limited sample size, and methodological constraints described in the Limitations section (Lines 372–387). The revised conclusions now emphasize feasibility and practical clinical applicability rather than definitive superiority over conventional imaging modalities.

**Response to Reviewer #2**

**Comment #1: Methodology & Reference Standard**
The procedures for the reference standard need clarification, particularly regarding how bone void cysts were incorporated and the specific lymph node size rules used.

**Response**
We appreciate this important comment regarding methodological clarity. The Methods section (Reference standard) has been revised to provide a clearer and more precise description of the diagnostic framework (Lines170–187).

Benign cystic bone lesions, including bone void cysts, were not predefined as a separate diagnostic category. Instead, bone lesions were classified dichotomously as metastatic or non-metastatic based on consensus interpretation using all available imaging modalities (DWIBS, bone scintigraphy, and CT) in conjunction with longitudinal clinical and imaging follow-up, consistent with a best valuable comparator approach.

We have also explicitly clarified that lymph nodes with a short-axis diameter ≥8 mm were defined as metastatic. Furthermore, histopathological findings from extended pelvic lymph node dissection were used as the reference standard in 14 patients. These revisions were made to improve transparency and reduce potential ambiguity in the reference standard definition (Lines185–187).

**Comment #2: “Whole-body” Scope**
The term “whole-body” is used, but the reported anatomic coverage appears incomplete.

**Response**
We thank the reviewer for highlighting this important issue. We have revised the Methods and Discussion sections to clearly define the anatomic coverage of DWIBS as extending from the skull base to the proximal femur (Lines140–141).

In this study, the term “whole-body” DWIBS was used in accordance with our institutional protocol, in which the standard acquisition range covers the skull base to the proximal femur rather than vertex-to–mid-thigh coverage. To avoid potential misunderstanding, we have explicitly clarified this definition in the Methods section.

We now also acknowledge in the Discussion that this coverage does not fully conform to the vertex-to–mid-thigh range recommended by the METastasis Reporting and Data System for Prostate Cancer (MET-RADS-P) guidelines and discuss this as a limitation, particularly with respect to lesions located outside the imaging field (Lines348–352 and 365–370). Specifically, missed lesions were located above the skull base and therefore outside the acquisition range. In addition, all relevant descriptions and tables have been revised to ensure full internal consistency of the reported imaging coverage throughout the manuscript.

**Comment #3: Reader Reliability**
Agreement between different readers (inter-reader reliability) is missing.

**Response**
We agree that inter-reader reliability is an important methodological consideration. In this retrospective study, all DWIBS images were interpreted in consensus by two experienced radiologists as part of routine clinical practice, and independent reader-specific assessments were not retained in the database. Therefore, formal inter-reader reliability analysis could not be performed.

This limitation has now been explicitly acknowledged in both the Methods and Limitations sections to improve transparency (Lines142–145 and 380–384).

**Comment #4: Protocol & Timing Transparency**
Reporting of imaging protocols and timing between DWIBS and reference standard scans is incomplete.

**Response**
We thank the reviewer for this valuable comment. The timing of imaging examinations was originally described in the Study design and population section, where all patients were reported to have undergone DWIBS, CT, and bone scintigraphy within two months prior to treatment initiation. To improve clarity and avoid potential misunderstanding, we have added an explicit cross-reference to this timing description in the Imaging protocols section (Lines145–147).

In addition, our institutional DWIBS protocol includes both diffusion-weighted imaging and accompanying coronal T2-weighted sequences. Image interpretation was based on integrated assessment of all acquired sequences rather than diffusion-weighted imaging alone. Lung metastases identified during the DWIBS examination were mainly recognized on the accompanying T2-weighted images rather than diffusion-weighted images alone, and this point has been clarified in the revised manuscript to prevent potential misinterpretation (Lins55–57 and 274–280).

**Comment #5: Supporting Claims (Cost-effectiveness)**
Claims regarding cost-effectiveness are not supported by data.

**Response**
We fully agree with the reviewer that cost-effectiveness and direct substitution for other advanced imaging modalities were not directly evaluated in this study. Therefore, we have removed unsupported statements regarding cost-effectiveness and economic superiority from the Discussion and Conclusions sections to avoid overinterpretation.

The revised manuscript now focuses on feasibility, accessibility, and potential complementary clinical roles of DWIBS combined with chest CT, rather than unsupported economic claims.

**Comment #6: Internal Consistency**
Inconsistencies between tables, figures, and text need to be resolved.

**Response**
We appreciate the reviewer’s careful reading. We have thoroughly re-reviewed the entire manuscript and corrected inconsistencies among the text, tables, and figures, including discrepancies in patient numbers, imaging ranges, and diagnostic performance metrics.

All numerical data (e.g., sensitivity, specificity, and case counts) were re-verified to ensure full consistency between the Results section and corresponding tables. Minor inconsistencies in descriptive text and terminology were also corrected to improve overall data integrity and internal coherence of the manuscript.

**Response to Editor**

**Methodological clarity**
We revised the Methods section to provide a clearer and more precise definition of the reference standard, including the role of the best valuable comparator, lymph node size criteria, and the handling of bone lesions. The anatomic coverage of DWIBS (skull base to proximal femur), imaging timing, and the limitation regarding the absence of inter-reader reliability analysis have also been explicitly clarified in the revised manuscript.

**Data availability statement**
In accordance with the PLOS ONE Data Policy, we revised the data availability statement to replace the author contact with a non-author institutional contact point (Department of Urology, Tokai University School of Medicine; urotokai@tokai.ac.jp).

**Ethics statement**
The ethics statement has been confined exclusively to the Methods section, and any duplicate statements in other sections were removed to ensure compliance with journal guidelines.

**Copyrighted figures**
Thank you for your comment regarding potential copyright issues in Figures 2 and 3.

We have carefully reviewed these figures and identified that the schematic illustration (Figure 2 panel C), which was included for explanatory purposes, could potentially raise copyright concerns. In the revised manuscript, this schematic has been removed and replaced with original figures consisting exclusively of patient-derived imaging data generated at our institution. All remaining figures are original and were created by the authors for this study.

We believe that the revised figures fully comply with the CC BY 4.0 license requirements of PLOS ONE. No previously published copyrighted material remains in the revised submission.
