# Supplementary material for: Efficacy and limitations of diffusion-weighted imaging with background suppression-based whole-body magnetic resonance imaging in high-risk prostate cancer staging: A retrospective study
Source: PLoS One. 2026 Jul 23;21(7):e0354601. doi: 10.1371/journal.pone.0354601 (PMC13395373; doi:10.1371/journal.pone.0354601)
Supplement: Peer review history

# Peer review: Decision Letter 0

**DOI:** 10.1371/journal.pone.0354601.r001

**Authors:** Carmelo Caldarella

3 Feb 2026

PONE-D-25-61259

Efficacy and Limitations of Diffusion-weighted Imaging with Background Suppression-Based Whole-Body Magnetic Resonance Imaging in High-risk Prostate Cancer Staging: A Retrospective Study

PLOS One

Dear Dr. Nakajima,

Thank you for submitting your manuscript to PLOS ONE. After careful consideration, we feel that it has merit but does not fully meet PLOS ONE’s publication criteria as it currently stands. Therefore, we invite you to submit a revised version of the manuscript that addresses the points raised during the review process.

==============================

**Methodological concerns to be addressed:**

- data on reference standard

- details on anatomic coverage and timing/reporting of scans

- report inter-reader reliability for DWIBS and data on cost-effectiveness of DWIBS

==============================

Please submit your revised manuscript by Mar 20 2026 11:59PM. If you will need more time than this to complete your revisions, please reply to this message or contact the journal office at plosone@plos.org. When you're ready to submit your revision, log on to https://www.editorialmanager.com/pone/ and select the 'Submissions Needing Revision' folder to locate your manuscript file.

Please include the following items when submitting your revised manuscript:

- A letter that responds to each point raised by the academic editor and reviewer(s). You should upload this letter as a separate file labeled 'Response to Reviewers'.
- A marked-up copy of your manuscript that highlights changes made to the original version. You should upload this as a separate file labeled 'Revised Manuscript with Track Changes'.
- An unmarked version of your revised paper without tracked changes. You should upload this as a separate file labeled 'Manuscript'.

-->If you would like to make changes to your financial disclosure, please include your updated statement in your cover letter. Guidelines for resubmitting your figure files are available below the reviewer comments at the end of this letter.

If applicable, we recommend that you deposit your laboratory protocols in protocols.io to enhance the reproducibility of your results. Protocols.io assigns your protocol its own identifier (DOI) so that it can be cited independently in the future. For instructions see: https://journals.plos.org/plosone/s/submission-guidelines#loc-laboratory-protocols. Additionally, PLOS ONE offers an option for publishing peer-reviewed Lab Protocol articles, which describe protocols hosted on protocols.io. Read more information on sharing protocols at https://plos.org/protocols?utm_medium=editorial-email&utm_source=authorletters&utm_campaign=protocols.

We look forward to receiving your revised manuscript.

Kind regards,

Carmelo Caldarella, Ph.D., M.D.

Academic Editor

PLOS One

Journal Requirements:

When submitting your revision, we need you to address these additional requirements.

1.Please ensure that your manuscript meets PLOS ONE's style requirements, including those for file naming. The PLOS ONE style templates can be found at

https://journals.plos.org/plosone/s/file?id=wjVg/PLOSOne_formatting_sample_main_body.pdf and

https://journals.plos.org/plosone/s/file?id=ba62/PLOSOne_formatting_sample_title_authors_affiliations.pdf

2. In the online submission form you indicate that your data is not available for proprietary reasons and have provided a contact point for accessing this data. Please note that your current contact point is a co-author on this manuscript. According to our Data Policy, the contact point must not be an author on the manuscript and must be an institutional contact, ideally not an individual. Please revise your data statement to a non-author institutional point of contact, such as a data access or ethics committee, and send this to us via return email. Please also include contact information for the third party organization, and please include the full citation of where the data can be found.

3. Your ethics statement should only appear in the Methods section of your manuscript. If your ethics statement is written in any section besides the Methods, please delete it from any other section.

4. We note that Figure 2 and 3 in your submission contain copyrighted images. All PLOS content is published under the Creative Commons Attribution License (CC BY 4.0), which means that the manuscript, images, and Supporting Information files will be freely available online, and any third party is permitted to access, download, copy, distribute, and use these materials in any way, even commercially, with proper attribution. For more information, see our copyright guidelines: http://journals.plos.org/plosone/s/licenses-and-copyright.

We require you to either (1) present written permission from the copyright holder to publish these figures specifically under the CC BY 4.0 license, or (2) remove the figures from your submission:

1. You may seek permission from the original copyright holder of Figure 2 and 3 to publish the content specifically under the CC BY 4.0 license.

We recommend that you contact the original copyright holder with the Content Permission Form (http://journals.plos.org/plosone/s/file?id=7c09/content-permission-form.pdf) and the following text:

“I request permission for the open-access journal PLOS ONE to publish XXX under the Creative Commons Attribution License (CCAL) CC BY 4.0 (http://creativecommons.org/licenses/by/4.0/). Please be aware that this license allows unrestricted use and distribution, even commercially, by third parties. Please reply and provide explicit written permission to publish XXX under a CC BY license and complete the attached form.”

Please upload the completed Content Permission Form or other proof of granted permissions as an "Other" file with your submission.

In the figure caption of the copyrighted figure, please include the following text: “Reprinted from [ref] under a CC BY license, with permission from [name of publisher], original copyright [original copyright year].”

2. If you are unable to obtain permission from the original copyright holder to publish these figures under the CC BY 4.0 license or if the copyright holder’s requirements are incompatible with the CC BY 4.0 license, please either i) remove the figure or ii) supply a replacement figure that complies with the CC BY 4.0 license. Please check copyright information on all replacement figures and update the figure caption with source information. If applicable, please specify in the figure caption text when a figure is similar but not identical to the original image and is therefore for illustrative purposes only.

5. If the reviewer comments include a recommendation to cite specific previously published works, please review and evaluate these publications to determine whether they are relevant and should be cited. There is no requirement to cite these works unless the editor has indicated otherwise.

6. Please review your reference list to ensure that it is complete and correct. If you have cited papers that have been retracted, please include the rationale for doing so in the manuscript text, or remove these references and replace them with relevant current references. Any changes to the reference list should be mentioned in the rebuttal letter that accompanies your revised manuscript. If you need to cite a retracted article, indicate the article’s retracted status in the References list and also include a citation and full reference for the retraction notice.

Additional Editor Comments:

Dear Authors, the manuscript has been analyzed in depth by the Reviewers and based on their comments and suggestions, some concerns should be addressed, with particular regard to methodological section (more precise definition of reference standard, details on anatomic coverage and timing/reporting of scans, inter-reader reliability for DWIBS and data on cost-effectiveness if available from your experience).

Waiting for your revised version

Best regards

[Note: HTML markup is below. Please do not edit.]

Reviewers' comments:

Reviewer's Responses to Questions

**Comments to the Author**

1. Is the manuscript technically sound, and do the data support the conclusions?

The manuscript must describe a technically sound piece of scientific research with data that supports the conclusions. Experiments must have been conducted rigorously, with appropriate controls, replication, and sample sizes. The conclusions must be drawn appropriately based on the data presented.

Reviewer #1: Yes

Reviewer #2: No

**********

2. Has the statistical analysis been performed appropriately and rigorously?

Reviewer #1: Yes

Reviewer #2: I Don't Know

**********

3. Have the authors made all data underlying the findings in their manuscript fully available?

The PLOS Data policy requires authors to make all data underlying the findings described in their manuscript fully available without restriction, with rare exception (please refer to the Data Availability Statement in the manuscript PDF file). The data should be provided as part of the manuscript or its supporting information, or deposited to a public repository. For example, in addition to summary statistics, the data points behind means, medians and variance measures should be available. If there are restrictions on publicly sharing data—e.g. participant privacy or use of data from a third party—those must be specified.

Reviewer #1: No

Reviewer #2: Yes

**********

4. Is the manuscript presented in an intelligible fashion and written in standard English?

PLOS ONE does not copyedit accepted manuscripts, so the language in submitted articles must be clear, correct, and unambiguous. Any typographical or grammatical errors should be corrected at revision, so please note any specific errors here.

Reviewer #1: Yes

Reviewer #2: Yes

**********

5. Review Comments to the Author

Please use the space provided to explain your answers to the questions above. You may also include additional comments for the author, including concerns about dual publication, research ethics, or publication ethics. (Please upload your review as an attachment if it exceeds 20,000 characters)

Reviewer #1: This is a relatively small retrospective clinical study on the usefulness of Diffusion imaging (DWIBS) combined with chest CT to stage high risk metastatic prostate cancer. The paper's conclusion that "DWI plus chest CT offers an effective and balanced staging...and is a feasible alternative to PSMA-PET" should include repeating several of the caveats given from the paragraph on Limitations of the study (beginning with Line 279)

Reviewer #2: This study tackles a significant and clinically relevant question about cancer staging, and the finding that DWIBS could offer a radiation-free alternative with performance comparable to CT/bone scintigraphy is very promising.

To strengthen the manuscript for publication, several major points need to be addressed:

Methodology & Reference Standard: The procedures for the reference standard need clarification, particularly regarding how Bone Void Cysts (BVCs) were incorporated and the specific lymph node size rules used. As described, these choices may introduce bias.

"Whole-Body" Scope: The term "whole-body" is used, but the anatomic coverage reported appears incomplete. Please clarify the exact coverage or adjust the terminology.

Reader Reliability: A key piece of information is missing, the agreement between different readers (inter-reader reliability). Reporting this is essential for validating the diagnostic consistency of DWIBS.

Protocol & Timing Transparency: The reporting of imaging protocols and the timing between DWIBS and reference standard scans is incomplete. Adding these details is crucial for reproducibility.

Supporting Claims: The discussion includes claims about the cost-effectiveness of DWIBS, but the current study design does not provide data to support this. These claims should either be supported with analysis or removed.

Internal Consistency: There are several inconsistencies between tables, figures, and text (including dates) that need to be resolved to ensure data integrity.

**********

6. PLOS authors have the option to publish the peer review history of their article (what does this mean?). If published, this will include your full peer review and any attached files.

If you choose “no”, your identity will remain anonymous but your review may still be made public.

**Do you want your identity to be public for this peer review?**  For information about this choice, including consent withdrawal, please see our Privacy Policy.

Reviewer #1: No

Reviewer #2: **Yes:** Mohammad Reza Fattahi

**********

[NOTE: If reviewer comments were submitted as an attachment file, they will be attached to this email and accessible via the submission site. Please log into your account, locate the manuscript record, and check for the action link "View Attachments". If this link does not appear, there are no attachment files.]

To ensure your figures meet our technical requirements, please review our figure guidelines: https://journals.plos.org/plosone/s/figures

You may also use PLOS’s free figure tool, NAAS, to help you prepare publication quality figures: https://journals.plos.org/plosone/s/figures#loc-tools-for-figure-preparation.

NAAS will assess whether your figures meet our technical requirements by comparing each figure against our figure specifications.

# Author comment: Author response to Decision Letter 1

**DOI:** 10.1371/journal.pone.0354601.r002

20 Mar 2026

Response to Reviewers

We thank the Editor and the Reviewers for their careful evaluation of our manuscript and for their constructive and insightful comments. We have revised the manuscript accordingly and believe that these revisions have substantially improved the methodological clarity, transparency, and internal consistency of the study. All changes have been incorporated into the revised manuscript, and the corresponding sections have been updated as indicated.

________________________________________

Response to Reviewer #1

Comment #1

This is a relatively small retrospective clinical study on the usefulness of DWIBS combined with chest CT to stage high-risk metastatic prostate cancer. The paper’s conclusion that “DWI plus chest CT offers an effective and balanced staging…” should include repeating several of the caveats given in the Limitations section.

Response

We sincerely thank the reviewer for this constructive comment. We fully agree that the conclusions should appropriately reflect the limitations inherent to this retrospective study.

Accordingly, we have revised both the Abstract (Lines1–26) and the Conclusions (Lines 353–359) section to temper the wording and explicitly reiterate the key limitations, including the retrospective design, limited sample size, and methodological constraints described in the Limitations section (Lines 336–351). The revised conclusions now emphasize feasibility and practical clinical applicability rather than definitive superiority over conventional imaging modalities.

________________________________________

Response to Reviewer #2

Comment #1: Methodology & Reference Standard

The procedures for the reference standard need clarification, particularly regarding how bone void cysts were incorporated and the specific lymph node size rules used.

Response

We appreciate this important comment regarding methodological clarity. The Methods section (Reference standard) has been revised to provide a clearer and more precise description of the diagnostic framework (Lines134–151).

Benign cystic bone lesions, including bone void cysts, were not predefined as a separate diagnostic category. Instead, bone lesions were classified dichotomously as metastatic or non-metastatic based on consensus interpretation using all available imaging modalities (DWIBS, bone scintigraphy, and CT) in conjunction with longitudinal clinical and imaging follow-up, consistent with a best valuable comparator approach.

We have also explicitly clarified that lymph nodes with a short-axis diameter ≥8 mm were defined as metastatic. Furthermore, histopathological findings from extended pelvic lymph node dissection were used as the reference standard in 14 patients. These revisions were made to improve transparency and reduce potential ambiguity in the reference standard definition (Lines149–151).

________________________________________

Comment #2: “Whole-body” Scope

The term “whole-body” is used, but the reported anatomic coverage appears incomplete.

Response

We thank the reviewer for highlighting this important issue. We have revised the Methods and Discussion sections to clearly define the anatomic coverage of DWIBS as extending from the skull base to the proximal femur (Lines104–105).

In this study, the term “whole-body” DWIBS was used in accordance with our institutional protocol, in which the standard acquisition range covers the skull base to the proximal femur rather than vertex-to–mid-thigh coverage. To avoid potential misunderstanding, we have explicitly clarified this definition in the Methods section.

We now also acknowledge in the Discussion that this coverage does not fully conform to the vertex-to–mid-thigh range recommended by the METastasis Reporting and Data System for Prostate Cancer (MET-RADS-P) guidelines and discuss this as a limitation, particularly with respect to lesions located outside the imaging field (Lines312–316 and 329–334). Specifically, missed lesions were located above the skull base and therefore outside the acquisition range. In addition, all relevant descriptions and tables have been revised to ensure full internal consistency of the reported imaging coverage throughout the manuscript.

________________________________________

Comment #3: Reader Reliability

Agreement between different readers (inter-reader reliability) is missing.

Response

We agree that inter-reader reliability is an important methodological consideration. In this retrospective study, all DWIBS images were interpreted in consensus by two experienced radiologists as part of routine clinical practice, and independent reader-specific assessments were not retained in the database. Therefore, formal inter-reader reliability analysis could not be performed.

This limitation has now been explicitly acknowledged in both the Methods and Limitations sections to improve transparency (Lines106–109 and 344–348).

________________________________________

Comment #4: Protocol & Timing Transparency

Reporting of imaging protocols and timing between DWIBS and reference standard scans is incomplete.

Response

We thank the reviewer for this valuable comment. The timing of imaging examinations was originally described in the Study design and population section, where all patients were reported to have undergone DWIBS, CT, and bone scintigraphy within two months prior to treatment initiation. To improve clarity and avoid potential misunderstanding, we have added an explicit cross-reference to this timing description in the Imaging protocols section (Lines109–111).

In addition, our institutional DWIBS protocol includes both diffusion-weighted imaging and accompanying coronal T2-weighted sequences. Image interpretation was based on integrated assessment of all acquired sequences rather than diffusion-weighted imaging alone. Lung metastases identified during the DWIBS examination were mainly recognized on the accompanying T2-weighted images rather than diffusion-weighted images alone, and this point has been clarified in the revised manuscript to prevent potential misinterpretation (Lins19–21 and 238–244).

________________________________________

Comment #5: Supporting Claims (Cost-effectiveness)

Claims regarding cost-effectiveness are not supported by data.

Response

We fully agree with the reviewer that cost-effectiveness and direct substitution for other advanced imaging modalities were not directly evaluated in this study. Therefore, we have removed unsupported statements regarding cost-effectiveness and economic superiority from the Discussion and Conclusions sections to avoid overinterpretation.

The revised manuscript now focuses on feasibility, accessibility, and potential complementary clinical roles of DWIBS combined with chest CT, rather than unsupported economic claims.

________________________________________

Comment #6: Internal Consistency

Inconsistencies between tables, figures, and text need to be resolved.

Response

We appreciate the reviewer’s careful reading. We have thoroughly re-reviewed the entire manuscript and corrected inconsistencies among the text, tables, and figures, including discrepancies in patient numbers, imaging ranges, and diagnostic performance metrics.

All numerical data (e.g., sensitivity, specificity, and case counts) were re-verified to ensure full consistency between the Results section and corresponding tables. Minor inconsistencies in descriptive text and terminology were also corrected to improve overall data integrity and internal coherence of the manuscript.

________________________________________

Response to Editor

Methodological clarity

We revised the Methods section to provide a clearer and more precise definition of the reference standard, including the role of the best valuable comparator, lymph node size criteria, and the handling of bone lesions. The anatomic coverage of DWIBS (skull base to proximal femur), imaging timing, and the limitation regarding the absence of inter-reader reliability analysis have also been explicitly clarified in the revised manuscript.

Data availability statement

In accordance with the PLOS ONE Data Policy, we revised the data availability statement to replace the author contact with a non-author institutional contact point (Department of Urology, Tokai University School of Medicine; urotokai@tokai.ac.jp).

Ethics statement

The ethics statement has been confined exclusively to the Methods section, and any duplicate statements in other sections were removed to ensure compliance with journal guidelines.

Copyrighted figures

Thank you for your comment regarding potential copyright issues in Figures 2 and 3.

We have carefully reviewed these figures and identified that the schematic illustration (Figure 2 panel C), which was included for explanatory purposes, could potentially raise copyright concerns. In the revised manuscript, this schematic has been removed and replaced with original figures consisting exclusively of patient-derived imaging data generated at our institution. All remaining figures are original and were created by the authors for this study.

We believe that the revised figures fully comply with the CC BY 4.0 license requirements of PLOS ONE. No previously published copyrighted material remains in the revised submission.

Attachment Submitted filename: PLOS Response to reviewer.docx

# Peer review: Decision Letter 1

**DOI:** 10.1371/journal.pone.0354601.r003

**Authors:** Carmelo Caldarella

24 Jun 2026

PONE-D-25-61259R1

Efficacy and Limitations of Diffusion-weighted Imaging with Background Suppression-Based Whole-Body Magnetic Resonance Imaging in High-risk Prostate Cancer Staging: A Retrospective Study

PLOS One

Dear Dr. Nakajima,

Thank you for submitting your manuscript to PLOS ONE. After careful consideration, we feel that it has merit but does not fully meet PLOS ONE’s publication criteria as it currently stands. Therefore, we invite you to submit a revised version of the manuscript that addresses the points raised during the review process.

==============================

**ACADEMIC EDITOR:**

**Remaining issues:**  data availability policy (provide an anonymized minimal dataset or specific legal justification); small sample size and consequent effect on performance metrics.

==============================

Please submit your revised manuscript by Aug 08 2026 11:59PM. If you will need more time than this to complete your revisions, please reply to this message or contact the journal office at plosone@plos.org. When you're ready to submit your revision, log on to https://www.editorialmanager.com/pone/ and select the 'Submissions Needing Revision' folder to locate your manuscript file.

Please include the following items when submitting your revised manuscript:

- A letter that responds to each point raised by the academic editor and reviewer(s). You should upload this letter as a separate file labeled 'Response to Reviewers'.
- A marked-up copy of your manuscript that highlights changes made to the original version. You should upload this as a separate file labeled 'Revised Manuscript with Track Changes'.
- An unmarked version of your revised paper without tracked changes. You should upload this as a separate file labeled 'Manuscript'.

If you would like to make changes to your financial disclosure, please include your updated statement in your cover letter. Guidelines for resubmitting your figure files are available below the reviewer comments at the end of this letter.

If applicable, we recommend that you deposit your laboratory protocols in protocols.io to enhance the reproducibility of your results. Protocols.io assigns your protocol its own identifier (DOI) so that it can be cited independently in the future. For instructions see: https://journals.plos.org/plosone/s/submission-guidelines#loc-laboratory-protocols. Additionally, PLOS ONE offers an option for publishing peer-reviewed Lab Protocol articles, which describe protocols hosted on protocols.io. Read more information on sharing protocols at https://plos.org/protocols?utm_medium=editorial-email&utm_source=authorletters&utm_campaign=protocols.

As the corresponding author, your ORCID iD is verified in the submission system and will appear in the published article. PLOS supports the use of ORCID, and we encourage all coauthors to register for an ORCID iD and use it as well. Please encourage your coauthors to verify their ORCID iD within the submission system before final acceptance, as unverified ORCID iDs will not appear in the published article. *Only*  the individual author can complete the verification step; PLOS staff *cannot*  verify ORCID iDs on behalf of authors.

We look forward to receiving your revised manuscript.

Kind regards,

Carmelo Caldarella, Ph.D., M.D.

Academic Editor

PLOS One

Journal Requirements:

If the reviewer comments include a recommendation to cite specific previously published works, please review and evaluate these publications to determine whether they are relevant and should be cited. There is no requirement to cite these works unless the editor has indicated otherwise.

Please review your reference list to ensure that it is complete and correct. If you have cited papers that have been retracted, please include the rationale for doing so in the manuscript text, or remove these references and replace them with relevant current references. Any changes to the reference list should be mentioned in the rebuttal letter that accompanies your revised manuscript. If you need to cite a retracted article, indicate the article’s retracted status in the References list and also include a citation and full reference for the retraction notice.

Additional Editor Comments:

Dear Authors, please address the remaining issues raised by the Reviewers: particularly regarding data availability policy (you should at least provide an anonymized minimal dataset as a Supplementary Excel/CSV file or provide a specific legal justification), small sample size and consequent effect on performance metrics.

Best regards

[Note: HTML markup is below. Please do not edit.]

Reviewers' comments:

Reviewer's Responses to Questions

**Comments to the Author**

1. If the authors have adequately addressed your comments raised in a previous round of review and you feel that this manuscript is now acceptable for publication, you may indicate that here to bypass the “Comments to the Author” section, enter your conflict of interest statement in the “Confidential to Editor” section, and submit your "Accept" recommendation.

Reviewer #2: All comments have been addressed

Reviewer #3: All comments have been addressed

**********

2. Is the manuscript technically sound, and do the data support the conclusions?

The manuscript must describe a technically sound piece of scientific research with data that supports the conclusions. Experiments must have been conducted rigorously, with appropriate controls, replication, and sample sizes. The conclusions must be drawn appropriately based on the data presented.

Reviewer #2: Yes

Reviewer #3: Partly

**********

3. Has the statistical analysis been performed appropriately and rigorously?

Reviewer #2: Yes

Reviewer #3: Yes

**********

4. Have the authors made all data underlying the findings in their manuscript fully available?

The PLOS Data policy requires authors to make all data underlying the findings described in their manuscript fully available without restriction, with rare exception (please refer to the Data Availability Statement in the manuscript PDF file). The data should be provided as part of the manuscript or its supporting information, or deposited to a public repository. For example, in addition to summary statistics, the data points behind means, medians and variance measures should be available. If there are restrictions on publicly sharing data—e.g. participant privacy or use of data from a third party—those must be specified.

Reviewer #2: Yes

Reviewer #3: No

**********

5. Is the manuscript presented in an intelligible fashion and written in standard English?

PLOS ONE does not copyedit accepted manuscripts, so the language in submitted articles must be clear, correct, and unambiguous. Any typographical or grammatical errors should be corrected at revision, so please note any specific errors here.

Reviewer #2: Yes

Reviewer #3: Yes

**********

6. Review Comments to the Author

Please use the space provided to explain your answers to the questions above. You may also include additional comments for the author, including concerns about dual publication, research ethics, or publication ethics. (Please upload your review as an attachment if it exceeds 20,000 characters)

Reviewer #2: (No Response)

Reviewer #3: General Comments:

I would like to commend the authors for their diligent work in revising this manuscript. The current version is significantly improved. The clarification regarding the limited anatomical coverage (skull base to proximal femur) effectively explains the missed skull metastases, and the revised conclusions now accurately reflect the exploratory nature of a retrospective study. However, before the manuscript can be deemed fully suitable for publication in PLOS ONE, a few critical issues concerning data transparency, statistical interpretation, and formatting remain to be addressed.

Major Comments:

- Data availability policy: in the revised Data Availability statement, you mention that data are not publicly available due to institutional policies on patient confidentiality. Please note that PLOS ONE has a strict open-data policy. A minimal dataset containing demographic parameters (e.g., Age, PSA level, Gleason Score) and binary diagnostic outcomes (Positive/Negative for CT, BS, DWIBS, and Reference Standard) for the 38 patients can be completely anonymized (by removing dates, exact patient IDs, etc.) eliminating any risk of re-identification. I encourage you to provide this anonymized minimal dataset as a Supplementary Excel/CSV file to fully comply with the journal's mandate, or provide a specific legal justification (beyond generic institutional policy) why a fully anonymized table cannot be shared.

- Sample size and Confidence Intervals (CIs):While you acknowledge the small sample size (N=38) in the limitations (Lines 372-373), the text in the Results and Discussion does not adequately reflect the statistical uncertainty this causes. Looking at Tables 3 and 4, the 95% CIs are wide (e.g., Sensitivity for LN metastasis: 64.3%, with a 95% CI ranging from 35.1% to 87.2%). I recommend adding a brief sentence in the Discussion explicitly stating that the small sample size resulted in wide confidence intervals, meaning that the specific performance metrics (like the 64.3% sensitivity) should be interpreted as estimates requiring validation in larger, adequately powered cohorts.Reference Standard and Incorporation

- Bias:The use of the Best Valuable Comparator (BVC) is an accepted and pragmatic approach when histopathology is unavailable. However, using the index tests (DWIBS, CT, BS) to establish the reference standard inherently introduces an "incorporation bias," which artificially inflates the apparent diagnostic accuracy (especially specificity). This specific epidemiological term ("incorporation bias") should be explicitly mentioned in the Limitations section to ensure full methodological transparency for the readers.

**********

7. PLOS authors have the option to publish the peer review history of their article (what does this mean?). If published, this will include your full peer review and any attached files.

If you choose “no”, your identity will remain anonymous but your review may still be made public.

**Do you want your identity to be public for this peer review?**  For information about this choice, including consent withdrawal, please see our Privacy Policy.

Reviewer #2: **Yes:** Mohammad Reza Fattahi

Reviewer #3: No

**********

[NOTE: If reviewer comments were submitted as an attachment file, they will be attached to this email and accessible via the submission site. Please log into your account, locate the manuscript record, and check for the action link "View Attachments". If this link does not appear, there are no attachment files.]

To ensure your figures meet our technical requirements, please review our figure guidelines: https://journals.plos.org/plosone/s/figures

You may also use PLOS’s free figure tool, NAAS, to help you prepare publication quality figures: https://journals.plos.org/plosone/s/figures#loc-tools-for-figure-preparation.

NAAS will assess whether your figures meet our technical requirements by comparing each figure against our figure specifications.

# Author comment: Author response to Decision Letter 2

**DOI:** 10.1371/journal.pone.0354601.r004

30 Jun 2026

We thank the Academic Editor and Reviewer #3 for their valuable comments. We have carefully revised the manuscript in accordance with all comments. Specifically, we have (1) provided an anonymized minimal dataset (S1 Dataset) and revised the Data Availability Statement, (2) added statements regarding the impact of the limited sample size and wide confidence intervals, (3) discussed the potential incorporation bias associated with the Best Valuable Comparator, and (4) corrected several data inconsistencies identified during final verification of the analytic dataset, including revision of the number of bone metastases according to the reference standard from 11 to 9, with corresponding updates to the diagnostic performance metrics and related text. Additional minor clarifications were also made in the Methods and Abstract. A detailed point-by-point response is provided in the uploaded "Response to Reviewers" document.

Attachment Submitted filename: renamed_734af.docx

# Peer review: Decision Letter 2

**DOI:** 10.1371/journal.pone.0354601.r005

**Authors:** Carmelo Caldarella

9 Jul 2026

Efficacy and Limitations of Diffusion-weighted Imaging with Background Suppression-Based Whole-Body Magnetic Resonance Imaging in High-risk Prostate Cancer Staging: A Retrospective Study

PONE-D-25-61259R2

Dear Dr. Nakajima,

We’re pleased to inform you that your manuscript has been judged scientifically suitable for publication and will be formally accepted for publication once it meets all outstanding technical requirements.

Within one week, you’ll receive an e-mail detailing the required amendments. When these have been addressed, you’ll receive a formal acceptance letter and your manuscript will be scheduled for publication.

An invoice will be generated when your article is formally accepted. Please note, if your institution has a publishing partnership with PLOS and your article meets the relevant criteria, all or part of your publication costs will be covered. Please make sure your user information is up-to-date by logging into Editorial Manager at Editorial Manager® and clicking the ‘Update My Information' link at the top of the page. For questions related to billing, please contact billing support.

If your institution or institutions have a press office, please notify them about your upcoming paper to help maximize its impact. If they’ll be preparing press materials, please inform our press team as soon as possible -- no later than 48 hours after receiving the formal acceptance. Your manuscript will remain under strict press embargo until 2 pm Eastern Time on the date of publication. For more information, please contact onepress@plos.org.

Kind regards,

Carmelo Caldarella, Ph.D., M.D.

Academic Editor

PLOS One

Additional Editor Comments (optional):

Reviewers' comments:

Reviewer's Responses to Questions

**Comments to the Author**

1. If the authors have adequately addressed your comments raised in a previous round of review and you feel that this manuscript is now acceptable for publication, you may indicate that here to bypass the “Comments to the Author” section, enter your conflict of interest statement in the “Confidential to Editor” section, and submit your "Accept" recommendation.

Reviewer #3: All comments have been addressed

**********

2. Is the manuscript technically sound, and do the data support the conclusions?

The manuscript must describe a technically sound piece of scientific research with data that supports the conclusions. Experiments must have been conducted rigorously, with appropriate controls, replication, and sample sizes. The conclusions must be drawn appropriately based on the data presented.

Reviewer #3: (No Response)

**********

3. Has the statistical analysis been performed appropriately and rigorously?

Reviewer #3: (No Response)

**********

4. Have the authors made all data underlying the findings in their manuscript fully available?

The PLOS Data policy requires authors to make all data underlying the findings described in their manuscript fully available without restriction, with rare exception (please refer to the Data Availability Statement in the manuscript PDF file). The data should be provided as part of the manuscript or its supporting information, or deposited to a public repository. For example, in addition to summary statistics, the data points behind means, medians and variance measures should be available. If there are restrictions on publicly sharing data—e.g. participant privacy or use of data from a third party—those must be specified.

Reviewer #3: (No Response)

**********

5. Is the manuscript presented in an intelligible fashion and written in standard English?

PLOS ONE does not copyedit accepted manuscripts, so the language in submitted articles must be clear, correct, and unambiguous. Any typographical or grammatical errors should be corrected at revision, so please note any specific errors here.

Reviewer #3: (No Response)

**********

6. Review Comments to the Author

Please use the space provided to explain your answers to the questions above. You may also include additional comments for the author, including concerns about dual publication, research ethics, or publication ethics. (Please upload your review as an attachment if it exceeds 20,000 characters)

Reviewer #3: (No Response)

**********

7. PLOS authors have the option to publish the peer review history of their article (what does this mean?). If published, this will include your full peer review and any attached files.

If you choose “no”, your identity will remain anonymous but your review may still be made public.

**Do you want your identity to be public for this peer review?**  For information about this choice, including consent withdrawal, please see our Privacy Policy.

Reviewer #3: No

**********

# Editor report: Acceptance letter

**DOI:** 10.1371/journal.pone.0354601.r006

**Authors:** Carmelo Caldarella

PONE-D-25-61259R2

PLOS One

Dear Dr. Nakajima,

I'm pleased to inform you that your manuscript has been deemed suitable for publication in PLOS One. Congratulations! Your manuscript is now being handed over to our production team.

At this stage, our production department will prepare your paper for publication. This includes ensuring the following:

* All references, tables, and figures are properly cited

* All relevant supporting information is included in the manuscript submission,

* There are no issues that prevent the paper from being properly typeset

You will receive further instructions from the production team, including instructions on how to review your proof when it is ready. Please keep in mind that we are working through a large volume of accepted articles, so please give us a few days to review your paper and let you know the next and final steps.

Lastly, if your institution or institutions have a press office, please let them know about your upcoming paper now to help maximize its impact. If they'll be preparing press materials, please inform our press team within the next 48 hours. Your manuscript will remain under strict press embargo until 2 pm Eastern Time on the date of publication. For more information, please contact onepress@plos.org.

You will receive an invoice from PLOS for your publication fee after your manuscript has reached the completed accept phase. If you receive an email requesting payment before acceptance or for any other service, this may be a phishing scheme. Learn how to identify phishing emails and protect your accounts at https://explore.plos.org/phishing.

If we can help with anything else, please email us at customercare@plos.org.

Thank you for submitting your work to PLOS ONE and supporting open access.

Kind regards,

PLOS ONE Editorial Office Staff

on behalf of

Dr. Carmelo Caldarella

Academic Editor

PLOS One
